# Interferon block to HIV-1 transduction in macrophages despite SAMHD1 degradation and high deoxynucleoside triphosphates supply

**DOI:** 10.1186/1742-4690-10-30

**Published:** 2013-03-11

**Authors:** Loic Dragin, Laura Anh Nguyen, Hichem Lahouassa, Adèle Sourisce, Baek Kim, Bertha Cecilia Ramirez, Florence Margottin-Goguet

**Affiliations:** 1 Inserm, U1016, Institut Cochin, 22 rue Méchain, Paris, 75014, France; 2 Cnrs, UMR8104, Paris, France; 3 Univ Paris Descartes, Paris, France; 4 Department of Microbiology and Immunology, University of Rochester Medical Center, 601 Elmwood Avenue, Rochester, NY, 14642, USA

**Keywords:** SAMHD1, Interferon, Restriction, HIV

## Abstract

**Background:**

Interferon-α (IFN-α) is an essential mediator of the antiviral response, which potently inhibits both early and late phases of HIV replication. The SAMHD1 deoxynucleoside triphosphate (dNTP) hydrolase represents the prototype of a new antiviral strategy we referred to as “nucleotide depletion”. SAMHD1 depletes dNTP levels in myeloid cells below those required for optimal synthesis of HIV viral DNA. HIV-2 and its SIVsm and SIVmac close relatives encode a protein termed Vpx, which counteracts SAMHD1. The potentiality of IFN-α to cooperate with nucleotide depletion has been poorly investigated so far. Here we wondered whether IFN-α affects SAMHD1 expression, Vpx-induced SAMHD1 degradation, Vpx-mediated rescue of HIV-1 transduction and the dNTP supply in monocyte-derived macrophages (MDMs).

**Results:**

IFN-α inhibited HIV-1 transduction in monocytes and in MDMs while SAMHD1 expression was not up-regulated. Vpx triggered SAMHD1 degradation in IFN-α treated cells, and weakly restored HIV-1 transduction from the IFN-α block. Vpx helper effect towards HIV-1 transduction was gradually inhibited with increasing doses of IFN-α. dNTP levels were not significantly affected in MDMs and CD4+ primary activated T lymphocytes by IFN-α and, in correlation with SAMHD1 degradation, restoration of dNTP levels by Vpx was efficient in MDMs treated with the cytokine. In contrast, IFN-α inhibited Vpx-mediated SAMHD1 degradation in THP-1 cells, where, accordingly, Vpx could not rescue HIV-1 transduction.

**Conclusion:**

Our results suggest that the early antiviral effect of IFN-α results from a mechanism independent of nucleotide depletion in MDMs. In addition, they indicate that the macrophage-like THP-1 cell line may provide a system to characterize an IFN-α-induced cell response that inhibits Vpx-mediated SAMHD1 degradation.

## Background

To establish infections *in vivo*, type 1 and type 2 human immunodeficiency viruses (HIV-1 and HIV-2) have to face powerful cellular defense mechanisms including both intrinsic and innate immunity [1,2]. In a simplistic view, intrinsic immunity refers to the existence of restriction factors that inhibit virus entry as soon as the virus enters into the host cell, while innate immunity refers to the establishment of an antiviral state mediated by the production of IFN-α. However both phenomena are tightly linked through at least the ability of IFN-α to stimulate the expression of restriction factors that counteract HIV, including Trim5α, apolipoprotein B mRNA-editing enzyme catalytic polypeptide-like 3 G (APOBEC3G) and tetherin/Bone marrow stromal cell antigen 2 (BST-2/CD317) [3-8].

The IFN-α response is the major host immune response against viruses. Not only IFN-α enhances the innate immune response through the upregulation of antiviral cellular factors, but also IFN-α enhances adaptive immune responses. Evidence of IFN-α activity has been found in HIV-1 infected patients *in vivo*, notably through the role of the plasmacytoid dendritic cells, which detect viral RNA by Toll-like receptor 7 and subsequently represent a major source of IFN-α production [9]. IFN-α inhibits many steps of the viral life cycle, including the early steps of HIV-1 infection by inhibiting the accumulation of HIV-1 cDNA in monocyte-derived macrophages (MDMs) and to a lesser extent in primary CD4+ T cells [10-19]. IFN-γ is also detected at high levels in HIV-1 infected patients but in contrast to IFN-α, which can be expressed by all cells, IFN-γ is secreted by specialized cells (T helper cells, cytotoxic T cells and NK cells) [20]. Few reports indicate that it may also be a player in the early steps of the viral life cycle [15,16].

Macrophages play an important role in HIV infection [21]. They are infected *in vivo* and contribute to viral dissemination and transmission to other targets such as CD4+ T lymphocytes [21-25]. They also constitute a viral reservoir, which contributes to the establishment of latency making them critical players in the pathogenesis and ideal targets for anti-HIV therapy. Remarkably, intracellular factors like the recently identified SAMHD1 restriction factor drastically impair HIV infection in myeloid cells including macrophages [26-29].

SAMHD1 was initially identified as the human ortholog of the mouse gene *Mg11*, which is induced by IFN-γ treatment in macrophages [30]. Whether IFN upregulates SAMHD1 expression in human myeloid cells is unclear. In monocytes, IFN-α was shown to induce SAMHD1 expression [31]. However, another report showed that SAMHD1 expression is not upregulated by IFN-α, β or γ in monocyte-derived dendritic cells or primary T lymphocytes [32]. SAMHD1 has been proposed to act as a negative regulator of the innate immune response, analogous to Trex-1 [33]. Strikingly, mutations in the genes encoding SAMHD1, Trex1 and RNase H2 are all associated with Aicardi Goutières syndrome, a rare inflammatory encephalopathy characterized by over-production of IFN-α [34,35].

SAMHD1 is a dGTP-dependent deoxynucleoside triphosphates (dNTP) hydrolase [27,36,37]. By reducing the pool of dNTP in myeloid cells, SAMHD1 inhibits the reverse transcription step [27,32,38,39]. This mechanism of nucleotide depletion highlights previous data showing how low dNTP concentrations in myeloid cells limit HIV-1 infection [40-42]. Viruses from both the HIV-1 and HIV-2/SIVsm lineages are sensitive to SAMHD1, but only the HIV-2/SIVsm lineage has developed a weapon to counteract this host defense factor, the Vpx auxiliary protein [26,28]. These last years, Vpx was recognized as a viral protein that counteracts the cellular environment during the early phases of infection [43-48] until its cellular target was identified as being SAMHD1 [26,28]. As for other viral auxiliary proteins, Vpx usurps the activity of the Cul4A-based ubiquitin ligase through the DCAF1 adaptor, to induce the degradation of SAMHD1 [26,28,49]. Nucleotide depletion appears as a powerful mechanism of defense in quiescent cells that do not replicate their nuclear DNA, including CD4+ quiescent T cells, where SAMHD1 also restricts HIV-1 infection [38,39].

Here we explore the possibility that the IFN-α response, on the one hand, and nucleotide depletion, on the other hand, cooperate to counteract the infection in the early steps of HIV-1 infection in MDMs. We present evidence that SAMHD1 does not specifically contribute to the early-IFN-α block towards HIV-1. Furthermore, our results suggest that IFN-α does not affect the intracellular levels of dNTP at doses that inhibit HIV-1 transduction.

## Results and discussion

### A limited role of SAMHD1 in the early antiviral block mediated by IFN-α

**Figure 1:**
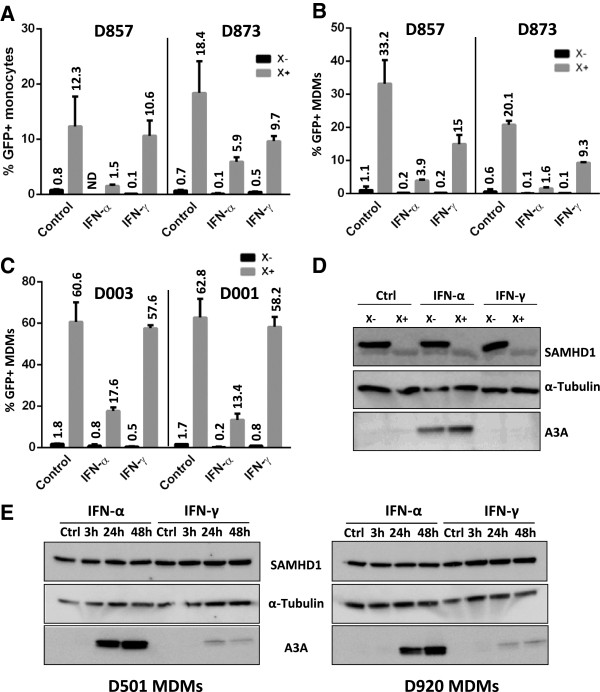
**Vpx poorly rescues HIV-1 transduction in IFN-α-primed monocytes/MDMs despite SAMHD1 degradation.** (**A**) Monocytes from two donors were either mock treated, or treated with IFN-α or IFN-γ (10000 U/ml). After 24 h, cells were incubated with empty or Vpx-containing VLP (X- and X+, respectively) for 2 h, and transduced with a VSV-G-pseudotyped GFP-encoding HIV-1 virus for 2 h at a multiplicity of infection (MOI) of 1. Three days post-transduction, the percentage of infected cells (% GFP + cells) was measured by flow cytometry. ND: not detected. (**B**) Same experimental procedure as in (**A**) performed with MDMs derived from the monocytes of the 2 donors presented in (**A**). (**C**) Same experimental procedure as in (**B**) performed in MDMs of 2 different donors, except that the concentration of IFN-α and IFN-γ has been reduced to 1000 U/ml. (**D**) SAMHD1 as well as APOBEC3A (A3A) protein levels were assessed by Western blot analysis from the cells in (**C**) two days post transduction (one representative Western blot is shown). (**E**): Western blot analysis of SAMHD1 and A3A expression in MDMs from two donors challenged or not (Ctrl) for the indicated periods with 1000 U/ml of IFN-α and IFN-γ.

**Figure 2:**
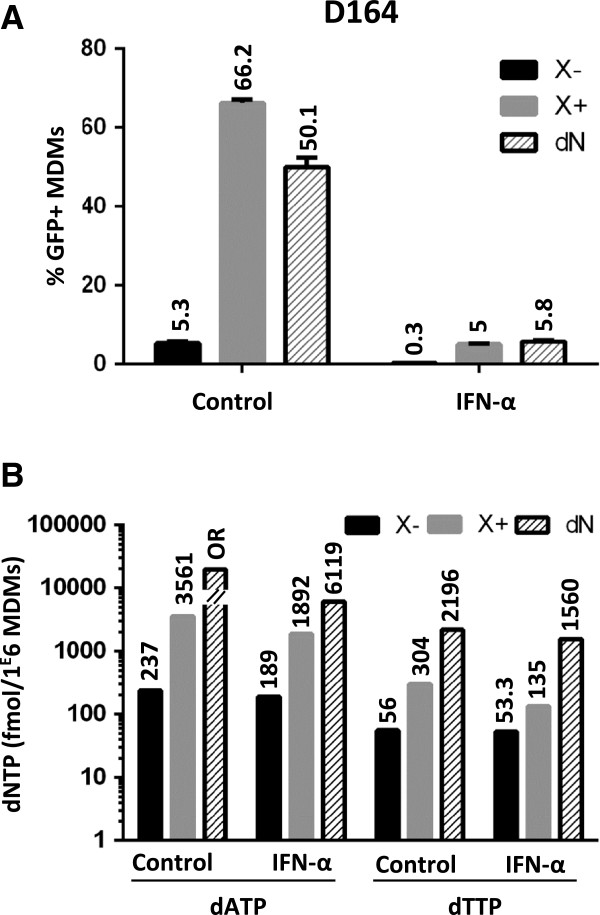
**The dNTP concentration in MDMs is not affected by IFN-α doses that inhibit HIV-1 transduction.** (**A**) MDMs transduction was carried out as in Figure 1A. Cells were treated or not with IFN-α (100 U/ml) for 24 h, then were incubated with empty or Vpx-containing VLP (X- and X+, respectively) for 2 h. Finally, the cells were transduced with a VSV-G-pseudotyped GFP-encoding HIV-1 virus for 2 h (MOI 1). Exogenous deoxynucleosides (dNs) were used as controls for further dNTP quantification. Three days post-transduction, the percentage of infected cells (% GFP + cells) was measured by flow cytometry. (**B**) dATP and dTTP levels were measured using the single nucleotide-incorporation assay (three donors, one representative donor data is shown). OR: out of range.

We reasoned that Vpx -which depletes SAMHD1- should rescue the early IFN-α block to HIV-1 transduction, if this block was the result of SAMHD1 activity. Monocytes (Figure 1A) or MDMs (Figure 1B) from two donors were treated for 24 hours with high doses of IFN-α or IFN-γ (10 000 units/ml), thereafter cells were incubated with empty or Vpx-containing viral-like particles (VLP X- or VLP X+) and infected with a GFP-encoding HIV-1 virus. The percentage of infected (GFP positive) cells was measured 3 days post-infection. HIV-1 transduction was inhibited by IFN-α in both cell types and to a lesser extent by IFN-γ though the respective effects of both IFN were difficult to assess because of the low transduction efficiencies. Vpx dramatically increased HIV-1 transduction as expected [45] and was able to slightly rescue HIV-1 transduction from the IFN-α inhibitory effects in correlation with SAMHD1 degradation (Figure 1A and B and Additional file 1: Figure S1). At lower doses of IFN-α, the rescue from the IFN-α block by Vpx was still weak (Figure 1C, 1000 U/ml; Figure 2A, 100 U/ml) despite undetectable levels of SAMHD1 (Figure 1D, 1000U/ml IFN-α or γ, one representative donor over three donors). Lower doses of IFN-γ did not significantly affect HIV-1 transduction or Vpx helper effect (Figure 1C), which led us to pursue our study only with IFN-α. Of note, APOBEC3A levels were efficiently increased by IFN-α as previously shown [50,51], and were not decreased by Vpx (Figure 1D). No change in SAMHD1 levels was observed in a time-course experiment where macrophages were treated with IFN-α or IFN-γ for different times, in contrast to APOBEC3A levels used as a control (Figure 1E).

**Figure 3:**
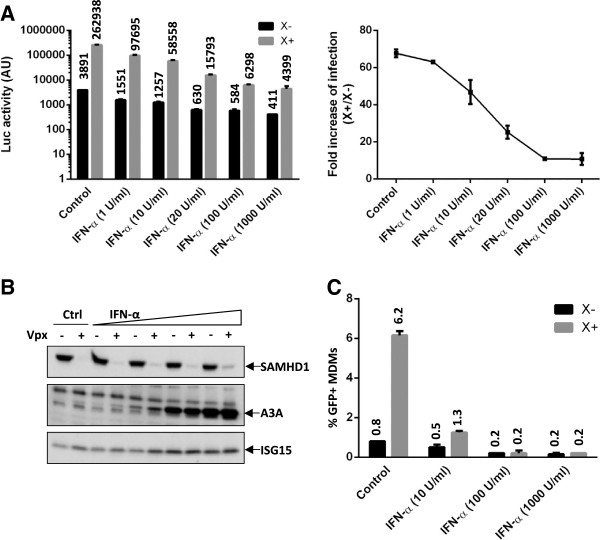
**Evidence that SAMHD1 does not contribute to the early IFN-α block to HIV-1 transduction.** (**A**) IFN-α inhibits Vpx helper effect toward HIV-1. MDMs were treated with increasing doses of IFN-α for 24 hours, incubated with empty or Vpx-containing VLP (X- or X+, respectively) for 2 h, then transduced with a VSV-G pseudotyped luciferase-encoding HIV-1 virus (HIV-1-Luc) normalized for infectivity, for 2 h. The transduction rate (Luc activity) was measured 2 days post-transduction (left panel). Vpx helper effect corresponds to the fold increase of luciferase activity between cells transduced with Vpx versus cells transduced without Vpx (right). The results shown are representative of three independent experiments. (**B**) Analysis of SAMHD1, APOBEC3A and ISG15 expression in MDMs treated as in (**A**). (**C**) Vpx is unable to rescue SIVmac from the IFN-α block. MDMs were treated with the indicated doses of IFN-α, incubated with empty or Vpx containing VLP (X- or X+, respectively) to trigger SAMHD1 degradation, before being transduced with a Vpx-deleted SIVmac-GFP reporter virus (MOI 1). The percentage of transduction (% GFP + cells) was measured by flow cytometry 3 days post-transduction. The bar graph is representative of the results obtained in two independent experiments with different MDMs donors.

These preliminary results argue for the existence of IFN-α induced anti-HIV interfering factors other than SAMHD1, but did not rule out a role of SAMHD1 in the early innate response in macrophages. In case SAMHD1 contributed to the IFN-α-block, we expected the helper effect of Vpx toward HIV-1 to be magnified under IFN-α treatment, which should be measurable by an enhancement of the transduction ratio “+ Vpx” over “- Vpx” (Vpx helper effect). Transduction efficiencies using HIV-1 GFP-expressing vectors were very low, namely in the presence of IFN-α, and precluded the determination of such ratios (Figure 1). We therefore transduced cells with a VSV-G pseudotyped HIV-1 luciferase virus, which enabled accurate quantification. Transduction was gradually inhibited with increasing doses of IFN-α, as well as the helper effect of Vpx toward HIV-1 transduction (Figure 3A). In parallel, SAMHD1 degradation was obtained at every single dose of IFN-α, while levels of APOBEC3A and ISG15, a second interferon-induced protein, were increased (Figure 3B). These results suggest that SAMHD1, which is well expressed at a basal level, is unlikely an early specific IFN-α-induced factor in macrophages. We further transduced IFN-α-primed macrophages with Vpx + VLP to trigger SAMHD1 proteasomal degradation and subsequently infected the cells with a Vpx-deleted GFP-encoding SIVmac virus, which is sensitive to SAMHD1 restriction. Unlike in control cells, where Vpx rescue was efficient, Vpx was unable to enhance SIV transduction in IFN-α treated cells even at low doses of IFN-α as previously reported (Figure 3C) [19,52,53].

Altogether, our results suggest that SAMHD1 does not specifically contribute to the IFN-α-block toward HIV-1 transduction in macrophages.

### The IFN-α block to HIV-1 transduction is not the result of a change in dNTP levels

We expected Vpx to enhance the dNTP pool to the same extent with or without IFN-α since SAMHD1 was degraded in both conditions. This assumption was directly explored by quantifying dNTP levels in MDMs treated or not with IFN-α. A 100U/ml dose of IFN-α was deliberately used to severely inhibit HIV-1 transduction without overwhelming the transduction pathway (Figure 2A). IFN-α did not affect the basal pool of intracellular dATP and dTTP, representative of the four dNTP (Figure 2B, one out of three representative donor data is shown). As expected, dATP and dTTP levels were drastically enhanced in the presence of Vpx or a 2 mM deoxynucleoside (dNs) treatment (Figure 2B). When cells were primed with IFN-α, neither Vpx nor dNs efficiently restored HIV-1 transduction (Figure 2A), though dNTP increase was still efficient in both conditions (Figure 2B). Thus, on the one hand, Vpx efficiently rescued dNTP levels under IFN-α treatment in correlation with SAMHD1 degradation despite poorly rescuing HIV-1 transduction. On the other hand, bypassing SAMHD1 dNTP hydrolase activity by providing dNs did not allow rescuing HIV-1 transduction from the IFN-α block (Figure 2A and B). Altogether, these results reinforce our conclusion that SAMHD1 does not contribute to the early interferon block to HIV-1 transduction and further suggest that dNTP levels are likely not targeted by IFN-α to establish this block in macrophages.

**Figure 4:**
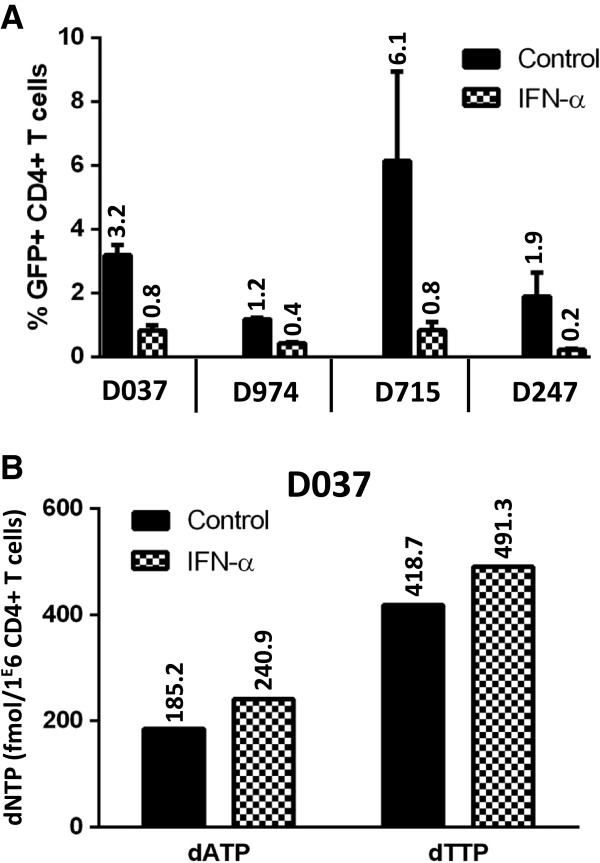
**dNTP levels in CD4+ activated T cells are unaffected by IFN-α.** (**A**) CD4+ activated T cells derived from 4 donors’ PBMC were either mock treated, or treated with a 1000 U/ml dose of IFN-α for 24 h. The cells were then challenged with a HIV-1-GFP reporter virus at MOI 4. The percentage of infection (% GFP + cells) was measured 3 days post-transduction by flow cytometry. (**B**) The intracellular dATP and dTTP levels were measured for one donor using the single nucleotide-incorporation assay.

Because activated CD4+ T lymphocytes contain a higher amount of dNTP than macrophages, we wondered whether IFN-α would affect the dNTP pool in primary T cells. HIV-1 transduction rates were lower in primary CD4+ T cells (Figure 4A) than in Jurkat or CEM T cell lines (respectively 83% and 40%, data not shown). A 1000 U/ml dose of IFN-α had a modest effect on the transduction rate, ranging from a 2 to 8-fold decrease (Figure 4A). Despite this reduction, dNTP levels were not affected (Figure 4B).

Altogether, our results suggest that nucleotide depletion does not contribute to the early IFN block toward HIV-1 transduction in macrophages or in activated CD4+ T lymphocytes.

### IFN-α inhibits Vpx-mediated SAMHD1 degradation in THP-1 cells

**Figure 5:**
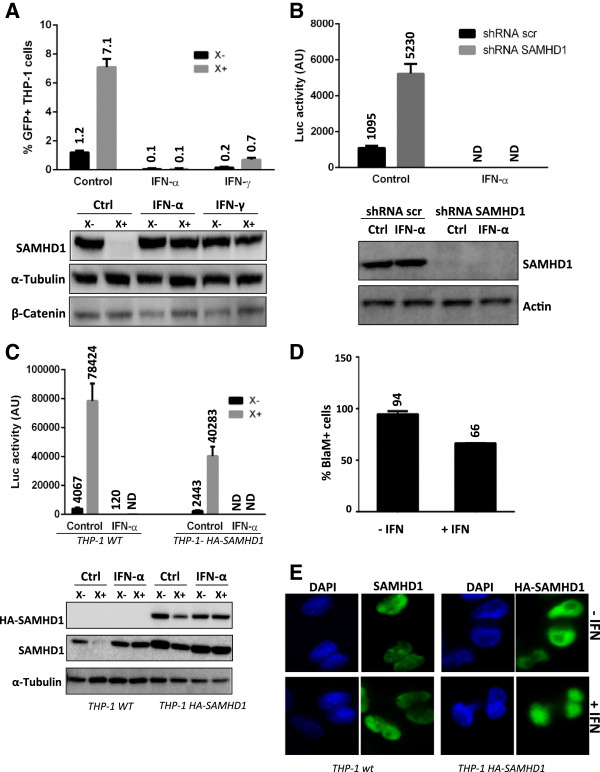
**Vpx-mediated SAMHD1 degradation is inhibited by IFNs in THP-1 cells.** (**A**) PMA-differentiated THP-1 cells were either mock treated, or treated with IFN-α or IFN-γ (1000 U/ml) during 24 h, then were incubated with empty or Vpx-containing VLP (X- and X+) for 2 h. Subsequently, cells were transduced with a VSV-G-pseudotyped GFP-encoding HIV-1 virus at MOI 1. The transduction rate was measured 3 days post-transduction (upper panel) and protein expression analyzed (lower panel). The results shown here are representative of three independent experiments. (**B**) SAMHD1-depleted THP-1 cells (shRNA SAMHD1) or control cells (shRNA scrambled scr) were treated with PMA, then primed with IFN-α (1000 U/ml) for 24 h, before transduction with a HIV-1-Luc reporter virus. The luciferase activity was measured 2 days post-transduction (upper panel) and SAMHD1 expression analyzed (lower panel). ND: not detected. (**C**) A monoclonal THP-1 cell line stably expressing HA-tagged SAMHD1 as well as wt THP1 cells were differentiated by PMA. Cells were primed with IFN-α (1000 U/ml) for 24 h, before incubation for 2 h with empty or Vpx containing VLP (X- or X+, respectively) and transduced with a HIV-1-Luc reporter virus. The luciferase activity was measured 2 days post-transduction (upper panel). Levels of endogenous and HA-tagged SAMHD1 were analyzed by western blot (lower panel). ND: not detected. (**D**) PMA-differentiated THP-1 cells were primed or not with IFN-α (10000 U/ml, 24 h). The BlaM-Vpr fusion entry test was then carried out as described in the methods. (**E**) IFN-α does not modify SAMHD1 nuclear localization. PMA-differentiated THP-1 cells expressing or not HA-SAMHD1 were primed with IFN-α (1000 U/ml) for 24 h. Cells were stained with the indicated antibodies for immunofluorescence (green). Nuclei were stained with DAPI (blue).

Phorbol 12-myristate 13-acetate (PMA)-treated THP-1 cells recapitulate the MDMs phenotype in many aspects and notably model MDMs in their sensitivity to Vpx-containing VLP and Vpx-deleted SIV viruses. Surprisingly, Vpx was unable to rescue HIV-1 transduction from the IFN-α-block in these cells (Figure 5A and C, top) unlike in primary MDMs. This lack of rescue correlated with the inability of Vpx to induce the degradation of SAMHD1 in IFN-α-treated THP-1 cells (Figure 5A and C, bottom). Similar results were obtained with IFN-γ. The levels of β-catenin, a very unstable protein, remained unchanged under IFN-α or IFN-γ treatments, suggesting that the inhibition of Vpx-mediated SAMHD1 degradation was not the result of an alteration of the proteasomal degradation pathway (Figure 5A, bottom). No rescue from the block was observed in SAMHD1-depleted THP-1 cells [28] further demonstrating that SAMHD1 did not contribute to the IFN block in THP-1 cells (Figure 5B). We further wondered why SAMHD1 was resistant to Vpx-mediated degradation in THP-1 cells. We asked whether this effect was the result of an inhibition of VLP entry using a β-lactamase-Vpr (BlaM-Vpr) virion-based fusion assay. This test consists in the use of viruses containing a BlaM-Vpr protein chimera. The delivery of BlaM-Vpr in the target cell is monitored by the enzymatic cleavage of a fluorogenic substrate of β-lactamase. 30% of decrease of viral entry was observed in the presence of very high doses of IFN-α but not at lower doses that remain sufficient to inhibit Vpx-mediated SAMHD1 degradation (Figure 5D and data not shown). Thus, a defect in viral entry was not responsible for the inhibition of Vpx-mediated SAMHD1 degradation in THP-1 cells. We further wondered whether an effect of IFN-α on the SAMHD1 promoter was at stake. To address this question, we stably transfected cells with a retroviral vector encoding HA-SAMHD1 under the control of the CMV promoter. Interestingly, expression of exogenous SAMHD1 in THP-1 cells, which already express endogenous SAMHD1, enhanced the restriction against HIV-1 by a 2-fold (Figure 5C, top). HA-SAMHD1 was degraded in the presence of Vpx and this process was inhibited by IFN-α (Figure 5C, bottom). This result suggests that inhibition of Vpx-mediated SAMHD1 degradation does not depend on an effect of IFN-α on the endogenous SAMHD1 promoter. Furthermore no change in the levels of DCAF1 or DDB1, subunits of the Cul4-based ubiquitin ligase used by Vpx, was observed under IFN treatment (data not shown). Finally, we asked whether IFN-α treatment could perturb SAMHD1 localization. Both endogenous SAMHD1 and the stably expressed HA-tagged-protein were present in the nucleus in agreement with previous reports [54-56] (Figure 5E). Priming with IFN-α did not perturb this localization.

## Conclusions

Here we explore the potential interplay between the role of IFN-α during the early steps of viral infection and the mechanism of nucleotide depletion governed by SAMHD1. We provide several lines of evidence that SAMHD1 is unlikely a major contributor of the early block imposed by IFN-α to HIV-1 transduction in MDMs, indeed: (i) Vpx poorly rescues HIV-1 transduction from the IFN-α block; (ii) Vpx helper effect is inhibited by increasing doses of IFN-α; (iii) SAMHD1 expression is not enhanced at IFN doses that inhibit HIV-1 transduction; (iv) Vpx does not rescue a Vpx-deleted SIV virus which is sensitive to SAMHD1 restriction; (v) dNTP levels are not significantly affected under IFN-α priming and are similarly increased by Vpx with or without IFN-α. In contrast to our results, previous data suggested that type I IFN magnified the effect of Vpx toward HIV-1 transduction in MDMs [52]. These previous findings suggested that the Vpx target was IFN-inducible and that Vpx, as for other viral auxiliary proteins, was able to escape some mechanisms of innate immunity. At this time, SAMHD1 was not discovered yet and whether SAMHD1 expression was increased under IFN treatment could not be checked. We do not understand why Vpx helper effect in MDMs is magnified in one report and inhibited in our experimental conditions. These discrepancies may result from the different procedures followed to prepare macrophages, which may lead to the expression of different potent antiviral factors that influence viral permissiveness. In any case, our results rule out a predominant role of SAMHD1, or at least of the SAMHD1 dNTP hydrolase activity, in the early IFN-α block to HIV-1 transduction. Indeed, despite no effect of IFN-α on dNTP levels or SAMHD1 expression, HIV-1 transduction was severely inhibited, highlighting the existence of other anti-HIV interfering factors that contribute to the interferon response [57]. The degradation of SAMHD1 does not preclude these host factors from counteracting HIV. A minor effect of SAMHD1 in the IFN block cannot however totally be discarded especially with the recent discovery of its nuclease activity on nucleic acid substrates [58].

Whether Vpx triggers the degradation of host factors other than SAMHD1 remains a subject of intense investigation. In addition to counteracting SAMHD1, Vpx has been reported to induce the degradation of APOBEC3A, a cellular factor known to exert its antiviral effect at the step of viral DNA accumulation in HeLa cells and in dendritic cells [59,60]. Here, in MDMs, we did not observe APOBEC3A degradation in the presence of Vpx, while SAMHD1 was degraded. As we were the only ones to deliver Vpx to the cell with the use of VLP before looking at APOBEC3A levels, it might be that only neo-synthetized Vpx is able to trigger APOBEC3A degradation and not virion-packaged Vpx. This could result for example from different levels of expression of Vpx from one condition to the other. Alternatively, the resistance of APOBEC3A to Vpx-induced degradation may also be a specificity of MDMs. Interestingly, our preliminary data futher suggest that APOBEC3A levels are upregulated when SAMHD1 is depleted, raising the possibility of a cooperation between the two restriction factors to counteract the infection (data not shown).

The existence of several targets of Vpx has also been suggested in monocyte-derived dendritic cells where Vpx was shown to rescue an IFN block that occurred after HIV-1 cDNA penetrated the nucleus [53]. Dendritic cells may express specifically an antiviral protein at higher levels than do MDMs.

The cellular factors that sustain the inhibition of HIV-1 transduction by IFN-α remain to be identified and the mechanisms deciphered. Keeping in mind that IFN was shown to affect cDNA accumulation in myeloid and CD4 primary T cells [10-18], we wondered whether treatment with exogenous IFN-α would result in a depletion of the nucleotide pool. Not only IFN-α had no measurable effect on the dNTP pool at doses that inhibit HIV-1 transduction, but also Vpx still increased the dNTP pool in cells treated with IFN-α. Therefore IFN-α does not appear to cooperate with nucleotide depletion in MDMs to counteract HIV. Rather SAMHD1 and the interferon pathway likely act in synergy to prevent the infection. Part of it could be explained by the fact that the dNTP pool has to be tightly regulated in the cell to avoid mutagenic or cytotoxic effects [61]. Nonetheless we have found that the dNTP intracellular pool is reduced at high doses of IFN-α (data not shown).

Surprisingly PMA treated-THP-1 cells model MDMs in their sensitivity to Vpx toward HIV-1 transduction but not in their ability to support Vpx-mediated SAMHD1 degradation under IFN priming. We have ruled out several hypotheses that could have explained these differences, namely a transcriptional effect of IFN-α from the SAMHD1 promoter, an effect of IFN-α on SAMHD1 localization or on Vpx delivery in THP-1 cells. Other hypothesizes should be explored, for instance the involvement of the recently identified splice variants of SAMHD1 [62] or the IFN-mediated upregulation of host factors specifically in THP-1 cells that would disrupt Vpx-mediated SAMHD1 degradation. The discovery of the mechanism at stake should help to decipher the regulation of SAMHD1 restriction activity.

## Methods

### Cells and cell culture

PBMCs from the blood of anonymous donors (obtained in accordance with the ethical guidelines of the Institut Cochin, Paris) were obtained by Ficoll density-gradient separation. Monocytes were isolated by positive selection on CD14 magnetic microbeads (Miltenyi Biotec) and CD4+ T cells were isolated by positive selection with CD4 magnetic microbeads (Milteny Biotec), followed by culture in BD Primaria flasks with R10 medium (RPMI-1640 GlutaMAX-I, 10 mM HEPES, pH 7.2, 1 mM sodium pyruvate, 1% (vol/vol) nonessential amino acids and 10% (vol/vol) heat-inactivated FCS) and antibiotics. MDMs were obtained from monocytes by culture of the cells for 7–9 d with granulocyte-macrophage colony-stimulating factor (10 ng/ml) and macrophage colony-stimulating factor (20 ng/ml). The primary CD4+ T cells were activated with phytohemaglutinin A (PHA-L; 3.0 mg/mL; Sigma-Aldrich) and interleukin-2 (IL2; 50 IU/mL; PeproTech) for 2 days prior to treatment with 1000 U/ml of IFN-α for 24 h. Human monocytic THP-1 cells and lymphoid Jurkat and CEM cells were maintained in R10 medium. THP-1 cells were differentiated with a 65 nM phorbol 12-myristate 13-acetate (PMA) treatment for 24 h. A THP-1-HA-SAMHD1 polyclonal cell line was obtained after cell transduction with viral particles expressing HA-SAMHD1 from the pLenti vector [27] and puromycin selection (2 μg/ml for 2 weeks). A monoclonal cell line was further developed by plating one cell expressing HA-SAMHD1 per well with the help of a flow cytometer. SAMHD1-depleted THP-1 cells were previously described [28].

### Viruses and virus-like particles (VLP) production

VLP and viruses were produced in 293 T cells co-transfected by the calcium-phosphate method according to Berger et al. [45,63]. Vpx-containing and control SIVmac VLP were produced by transfection of 293 T cells with pSIV3^+^ Δ*vpr* (VLPs X^+^) or pSIV3^+^ Δ*vpx* Δ*vpr* (VLP X^−^) [52] along with a plasmid encoding for vesicular stomatitis virus glycoprotein (VSV-G) in a ratio of 10:1. For the SIVmac-GFP reporter virus, the minimal SIVmac genome pGAE 1.0, in which GFP expression is driven from the cytomegalovirus promoter, was added to the packaging and VSV-G encoding plasmids in a ratio of 5:5:1 [45,63]. The HIV-1-GFP and HIV-1-Luc viruses were produced by transfection of cells with pRRLsin.eGFP and pCTS.Luc plasmids, respectively, with pCMVΔR8.91 packaging and VSV-G plasmids, in a ratio of 5:5:2 [45,63,64]. β-lactamase-Vpr (BlaM-Vpr) containing particles were produced by transfection of cells with the pCMV-BlaM-Vpr plasmid along with the pCMVΔR8.91 packaging and the VSV-G encoding vectors [65,66]. The culture supernatants were collected 48 h and 72 h after transfection and passed through 0.45-μm pore filters. The viral particles were then concentrated in 10% polyethylene glycol 6000 (PEG-6000) (Sigma) containing 300 mM NaCl and titrated by quantification of HIV-1 p24 and SIV p27 capsid using an enzyme-linked immunosorbent assay (ELISA) (ZeptoMetrix Corp.). Blam-Vpr containing particles were concentrated by ultracentrifuged through a 25% sucrose cushion in TNE buffer (100 mM NaCl, 10 mM Tris-HCl, pH 7.4, and 1 mM EDTA) at 150000 X g for 1 h. All viruses were normalized by single-cycle infectivity assays on HeLa cells.

### Transductions

Cells were transduced as described before for infection of dendritic cells [63]. Cells were preincubated with VLP for 2 h, then, when required, transduced with the indicated virus. The minimal amount of VLP (X^+^) required for maximal helper effect for HIV-1 infection was determined by functional titration. After preincubation, cells were infected for 2 h with the indicated reporter virus at a multiplicity of infection (MOI) of 1. Where indicated, infected cells were treated for 20 h with deoxynucleosides, which consisted of a mixture of dA (D8668), dC (D0776), dG (D0901) and dT (T1895; all from Sigma-Aldrich) as previously described [27].

### Western blot procedure and antibodies

Cells were lysed in adequate volumes of M-PER buffer (Pierce) containing 150 mM NaCl and an anti-protease mixture (Sigma) [67]. Protein extracts were separated by SDS-PAGE electrophoresis, transferred onto PVDF membranes, and revealed by a chemiluminescence procedure (CDP*Star*®, Applied Biosystems). Signals were acquired by a LAS 3000 apparatus (Fujifilm) for further analysis using the Multigauge software (Fujifilm). The following antibodies were used: monoclonal anti-HA tag antibody [16B12], anti-SAMHD1 [1 F9], and anti-ISG15 [3E5] from Abcam; monoclonal anti-α-Tubulin and anti-Actin from Sigma, monoclonal anti-β-Catenin from BD Bioscience, and polyclonal anti-A3G Apo-C17 antibody that recognizes both A3G and A3A (recognized for its smaller size, [59]) from the AIDS Reagents and Reference Program of the NIH.

### Quantification of whole-cell dNTP pools

The dNTP were quantified as previously described [40]. Briefly, cells were harvested and lysed in cold 65% aqueous methanol, vortexed vigorously, and heated to 95°C for 5 min. Cellular debris were removed from the sample through centrifugation at 13,000 rpm for 3 min. Supernatant was removed and dried in a speed vacuum. The dried samples were suspended in (50 mM Tris-HCl, pH 8.0, and 10 mM MgCl_2_)_._ Extracts were incubated with 200 fmol of primer/template. An 18-nucleotide primer labeled at the 5^′^ end with 32 P (5^′^-GTCCCTGTTCGGGCGCCA-3^′^) was annealed at a 1:2 ratio to four different 19-nucleotide templates (5^′^-NTGGCGCCCGAACAGGGAC-3^′^), where ‘N’ represents dG, dC, dA or dC. The reactions contained 2 μl of dNTP cell extract, 4 μl of excess HIV-1 RT, 25 mM Tris–HCl, pH 8.0, 2 mM dithiothreitol, 100 mM KCl, 5 mM MgCl2, and 10 μM oligo(dT) to a final volume of 20 μL. Reactions were incubated for 5 min at 37°C and terminated using 10 μl 40 mM EDTA, 99% formamide and heated at 95°C for 5 min. For analysis, reaction products were separated on a 14% polyacrylamide-urea denaturing gel (SequaGel, National Diagnostics) and analyzed on a PhosphorImager (PerkinElmer). Product extension was quantified by densitometry (Quantity One) and dNTP amount was calculated based on accountability of the dNTP sample dilution factor, so that each sample volume was adjusted to obtain a signal within the linear range of the assay’s standard curve.

### BlaM-Vpr entry test

The BlaM-Vpr entry test was performed as described in [65] by measuring enzymatic cleavage of a β-lactamase substrate (CCF2/AM, Invitrogen) loaded in target cells. Briefly, cells were incubated with HIV-1 VLP containing the BlaM-Vpr fusion protein for 2 h at 37°C. Cells were then incubated in CO2-independent medium containing the CCF2/AM β-lactamase substrate at room temperature in the dark for 12 hours before fixation and analysis by flow cytometry.

### Immunofluorescence

THP-1 cell monolayers were differentiated with PMA for 24 h on coverslips, then treated or not with 1000 U/ml IFN-α for 24 h. After washing and fixing as decribed in [68], cells were permeabilized for 3 + 15 min with PBT buffer (0.2% Tween 20, 1% bovine serum albumin in PBS). Subsequently, cells were incubated for 1 h at room temperature with primary antibodies in PBT buffer (Alexa Fluor® 488 anti-HA antibody (Invitrogen), or anti-SAMHD1 antibody [3 F5] (ab119751, Abcam). After three washes with PBS, when required, the cells were incubated for 30 min with secondary antibodies. Nuclei were stained with DAPI in mounting media (Vectashield; Vector Laboratories). Images were collected on a Zeiss Axio Observer.Z1 microscope using a 100X oil immersion objective, and analyzed with Metamorph 7 (Molecular Devices).

## Competing interests

The authors declare that they have no competing interests.

## Authors’ contributions

LD, HL, BCR and FMG conceived the study. LD, LAN, HL, AS, BCR and BK performed the experiments and/or participated in the experimental design. LD, BCR and FMG wrote the manuscript. All authors approved the final manuscript.

## Supplementary Material

Additional file 1: Figure S1. Western blot analysis of SAMHD1 expression in monocytes and MDMs (from Figure 1A and B) was carried out 3 days after IFN priming (one representative Western blot is shown for each cell type). (PDF 1046 kb) Click here for file
