# Supplementary figures and images for: Interferon block to HIV-1 transduction in macrophages despite SAMHD1 degradation and high deoxynucleoside triphosphates supply
Source: Retrovirology. 2013 Mar 11;10:30. doi: 10.1186/1742-4690-10-30 (PMC3599726; doi:10.1186/1742-4690-10-30)

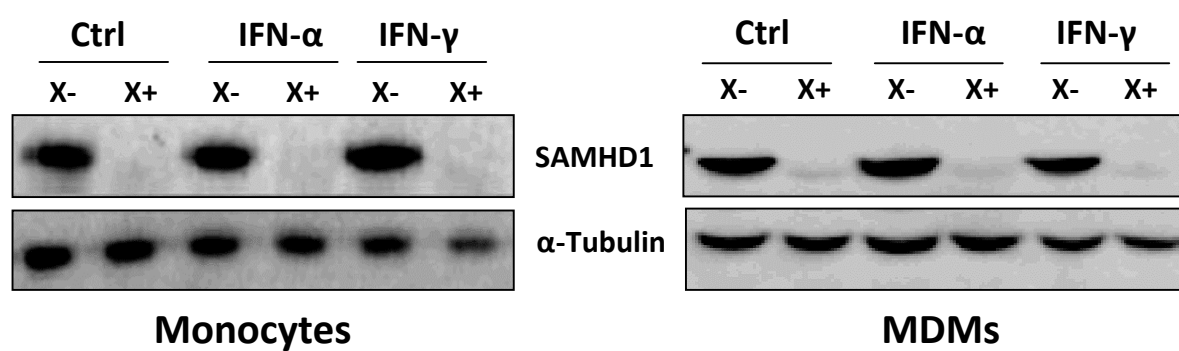

Dragin *et al.*, Supplementary figure 1

Supplement: Additional file 1: Figure S1. — Western blot analysis of SAMHD1 expression in monocytes and MDMs (from Figure 1A and B) was carried out 3 days after IFN priming (one representative Western blot is shown for each cell type). (PDF 1046 kb) [file 1742-4690-10-30-S1.pdf]
